# Use of Formulation Information Risk Management in Low Secure Services: An Audit Project

**DOI:** 10.1192/bjo.2022.431

**Published:** 2022-06-20

**Authors:** Alex Burns

**Affiliations:** South West Yorkshire Partnership NHS Foundation Trust, Wakefield, United Kingdom

## Abstract

**Aims:**

Risk assessment is a key component of patient care in forensic psychiatry. This audit aimed to measure the completion of different aspects of the Formulation Information Risk Management (FIRM) risk assessment for patients in the care of Low Secure Services. The FIRM incorporates formulation and a care plan into the risk assessment and should be completed for all inpatients in the trust. It was hoped that this audit would help identify any areas of improvement required in the completion of this risk assessment, and provide recommendations that would contribute to improving standards where required.

**Methods:**

Data were collected on 23rd December 2021 from the electronic patient records of 37 inpatients at a Low Secure Services Unit in Northern England. 5 audit criteria were devised following review of the trust standards regarding the completion of the FIRM assessment. These criteria included the completion of the Current / Historical Risks section, Formulation and Staying Safe / Staying Well Care Plan aspects of the assessment. It also assessed patient involvement in completion of the assessment and whether the assessment had been updated in the last Care Programme Approach (CPA) period. The findings of the audit were presented at a local academic meeting and were distributed to the relevant staff.

**Results:**

100% of patients had the Current / Historical Risks section completed

89% of the patients had the Formulation completed

73% of patients had the Staying Safe / Staying Well Care Plan completed

In 16% the service user had been involved in the risk assessment completion

In 70% of cases the FIRM had been updated since the last CPA (or in the last year if not applicable)

**Conclusion:**

Current / Historical Risks section completion rates matched expected trust standards. Significant improvement was seen in completion of the Formulation and Care Plan compared to auditing done in October 2021. There was room for improvement regarding increasing patient involvement in the completion of the risk assessment, often due to it being completed at night leading to the patient being unavailable. It was recommended that the FIRM should be more consistently reviewed and updated as part of each patient's 6 monthly CPA review. A re-audit would prove useful to monitor the progress of these measures, and the scope of a future audit could also be widened to include the timeliness in which the FIRM is completed for new patients.

